# IL-36 cytokines in autoimmunity and inflammatory disease

**DOI:** 10.18632/oncotarget.22814

**Published:** 2017-12-01

**Authors:** Liping Ding, Xiaohui Wang, Xiaoping Hong, Liwei Lu, Dongzhou Liu

**Affiliations:** ^1^ Department of Rheumatology and Immunology, Shenzhen People's Hospital, The Second Clinical Medical College of Jinan University, Shenzhen, China; ^2^ Department of Pathology and Shenzhen Institute of Research and Innovation, The University of Hong Kong, Hong Kong, China

**Keywords:** interleukin-36, inflammatory disease, psoriasis, arthritis

## Abstract

The inteleukin-36 (IL-36) cytokines include IL-36α, IL-36β, IL-36γ and IL-36Ra, which belong to the IL-1 family and exert pro-inflammatory effects on various target cells such as keratinocytes, synoviocytes, dendritic cells and T cells. Emerging evidence has suggested a role of IL-36 in the pathogenesis of many inflammatory diseases. Here, we provide a brief review on the activation of IL-36 family cytokines and their involvement in autoimmunity and inflammatory diseases, which will provide further insights in understanding the functions of IL-36 family cytokines in the pathophysiology of autoimmunity and inflammatory diseases.

## INTRODUCTION

Interleukin (IL)-36, a novel member of IL-1 cytokine family, was initially identified by the DNA database screens searching for homologs to IL-1 in 1999. Upon the elucidation of its biological function, each of these IL-1 family members has been assigned an individual interleukin designation [1]. IL-36 cytokines consist of four members, with IL-36α, IL-36β, and IL-36γ acting as IL-36 receptor agonists, while IL-36 receptor antagonist (Ra) is a cytokine that inhibits the activation of IL-36 receptor (IL-36R) signaling. IL-36 cytokines bind to the IL-36R and use the IL-1 receptor accessory protein (IL-1RAcP) as a co-receptor. Functionally, IL-36 cytokines exert pro-inflammatory effects, with certain differential functions depending on the type and site of inflammation. Since their abundant expression in keratinocytes (KCs) of skin and correlation with Th1 and Th17 cytokines in human psoriatic skin lesions, IL-36 cytokines have been best characterized in models of psoriasis [2]. Over the past years, the pathological effects of IL-36 family cytokines have been extended to a variety of inflammatory diseases, including psoriatic arthritis (PsA), systemic lupus erythematosus (SLE), inflammatory bowel disease (IBD), ulcerative colitis and Crohn's disease [1–6]. Furthermore, IL-36 cytokines also respond to various inflammatory signals induced by microbial infections, suggesting a role of these cytokines in anti-microbial inflammation [7]. In this review, we aim to discuss recent research on the role and mechanisms of IL-36 in regulating the immunity and inflammatory disease, which may provide the rationale for the development of IL-36-targeted therapies for treating psoriasis and other inflammatory diseases.

### IL-36 processing and secretion

The IL-36 cytokines are expressed in a variety of cell types, with abundant expression in KCs, bronchial epithelium, neuron cells, glial cells, dendritic cells (DCs) and macrophages, suggesting an important role of IL-36 cytokines in the homoeostasis and inflammation of the related tissues [2–4]. The activities of the N-terminal domain truncated IL-36α, IL-36β and IL-36γ increased by more than 1000-fold in comparison to their non-mutant forms [8]. The protein structure surrounding the truncation sites resembles proteases site other than caspase-1 cleaving site. Recently, Cathepsin S was demonstrated as the major IL-36γ-activating protease expressed by barrier tissues [9]. Several studies have reported that epidermal growth factor activates the predominant production of IL-36α and IL-36β in the skin, indicating the contribution of IL-36 cytokines in the skin homeostasis [10]. Stimulations with ligands for TLR2 and TLR4 such as LPS from *Escherichia Coli* and *Porphyromonas gingivalis* induce significant production of IL-36γ, TNFα, IL-6 and IL-32 in a human monocytic cell line (THP-1), and revealing an involvement of IL-36 in regulating innate immunity [11]. In various models of asthma, IL-36 cytokines are produced by lung epithelial cells in response to various inflammatory stimuli [12–13]. Moreover, peripheral blood lymphocytes are able to express IL-36γ upon stimulation of α-particles whereas T lymphocytes have been reported to express the IL-36 agonists under specific conditions [14]. Notably, IL-36α is upregulated in synovium-infiltrated plasma cells of PsA and rheumatoid arthritis (RA) patients, linking plasma cells to inflammatory cytokine production [15]. In cultured human KCs, IL-36 cytokines can also induce the expression of themselves in an autocrine loop similar to IL-1, underscoring its potent proinflammatory property [16]. Together, these findings indicate that IL-36 cytokines are secreted in a stimulus-dependent manner comparable to the induction of other IL-1 cytokines. However, the regulatory mechanisms underlying the production of IL-36 cytokines under different conditions still remain to be elucidated.

### IL-36 receptor and signaling pathway

IL-1F5, IL-1F6, IL-1F8, and IL-1F9, which are now designated as IL-36Ra, IL-36α, IL-36β, and IL-36γ, respectively. These four novel members, collectively called IL-36 cytokines, are part of the IL-1 family [17]. The encoding genes of these cytokines are located on the human chromosome 2q13 within 360kb region [13–14]. All these cytokines bind to IL-1 receptor (IL-1R)-related protein2. Agonistic IL-36R ligation by IL-36α, IL-36β, or IL-36γ leads to the recruitment of IL-1RAcP, the common accessory protein of IL-1, resulting in the activation of signaling pathway similar to those induced by IL-1α and IL-1β [16]. IL-36γ is reported to activate NF-κB in Jurkat cells in an IL-36R-dependent manner [18]. Subsequent studies have shown that IL-36α, IL-36β and IL-36γ, bind to IL-1Rrp2 and employ IL-1RAcP as a co-receptor in Jurkat cells as well as in several other human and mouse cell lines [19].

IL-36Ra, sharing more than 50% homology with IL-1 receptor antagonist (IL-1Ra), has been reported to elicit its antagonist activity through binding to the IL-36R and preventing the recruitment of IL-1RAcP [17]. Animal studies using gene deficient mice show that the anti-inflammatory effect of IL-36Ra on LPS-induced IL-1β is dependent on the IL-4 induction and recruitment of the anti-inflammatory SIGIRR/TIR8 [20]. These findings suggest that IL-36Ra plays anti-inflammatory role *via* its specific signaling pathway. Since IL-1Ra has not been found to induce cytokine production, IL-36Ra acts differently from the classical antagonist IL-1Ra.

The IL-36R is expressed by various cell types, including KCs, monocytes, DCs, and CD4^+^ T lymphocytes [13]. Published studies identifying the corresponding receptor and expression cells of the IL-36 cytokines are summarized in Table 1. Recent studies have revealed that human KCs contain IL-1RAcP. Notably, significant positive correlations have been observed among the protein expression of IL-36 cytokines with phosphorylated p38 MAPK and NF-κB p65 in psoriatic skin lesions. Moreover, the increased expression of IL-36 cytokines in psoriatic skin lesions may further amplify the activation of MAPK and NF-κB pathways [17, 21], indicating that IL-36 cytokines, similar to other IL-1 family members, can activate the MAPK and NF-κB pathways by binding to the corresponding receptor, IL-1RAcP.

**Table 1:** An overview of the IL-36 cytokines

IL-36 cytokines	Alternative names	Corresponding receptor	Expression cells	References
IL-36α	IL-IF6	IL-1Rrp2 and IL-1RAcP	Monocytes, T/B-lymphocytes,	[22–24]
IL-36β	IL-IF8	IL-1Rrp2 and IL-1RAcP	Monocytes, T/B-lymphocytes	[25–27]
IL-36γ	IL-IF9	IL-1Rrp2and IL-1RAcP	keratinocytes, epithelial cells	[28–30]
IL-36Ra	IL-IF5	Binds IL-1Rrp2 to SIGIRR	keratinocytes, Monocytes, DCs	[16, 22, 31]

### The biological functions of IL-36 family cytokines

The IL-36 agonists, IL-36α, IL-36β and IL-36γ, bind to the same receptor complex (IL-1Rrp2 and IL-1RAcP) to initiate proinflammatory signal transduction through the IL-36R. However, the biological functions of the IL-36 agonists are negatively regulated by IL-36Ra [29, 32]. IL-36 cytokines are found in KCs, bronchial epithelium, brain tissue, monocytes and macrophages [4]. Additionally, IL-36R responds to IL-36 agonists and is constitutively expressed by both bone marrow-derived dendritic cells (BMDCs) and CD4^+^ T lymphocytes, which have been shown as the major cell targets of IL-36 [10]. Emerging evidence indicates that IL-36 signaling is involved in the activation of innate and adaptive immune responses. DCs play a critical role in modulating the balance between immune tolerance and autoimmune inflammation, bridging the innate and adaptive arms of the immune system. Importantly, DCs possess the potent capacity in priming helper T (Th) cell differentiation. In murine DCs, IL-36 agonists upregulate maturation-associated CD80, CD86 and MHCII and induce the production of IL-12, IL-1β, IL-6, TNF-α and IL-23 in an IL-36R-dependent fashion. IL-36 agonists also enhance IL-36R-expressing CD4^+^ T cells to produce IFN-γ, IL-4, and IL-17A in a dose dependent manner [22]. The interaction between Th1 cytokines and IL-36 has also been well demonstrated in murine CD4^+^ T cells. IL-36 not only induces the production of IFN-γ in cultured splenocytes and activated CD4^+^ T cells, but also acts as an adjuvant to stimulate type 1 T helper (Th1) response in mice intradermally immunized using BSA [22]. Moreover, IL-36 agonists can directly polarize the differentiation of IFN-γ-producing Th1 cells [33]. The direct induction of IL-17A by IL-36 agonists is observed in cultured murine CD4^+^ T cells [22]. It has been shown that TNF-α and Th17 cytokines such as IL-17A and IL-22 directly induce IL-36 cytokines and, in turn, enhance their own expression and the production of proinflammatory cytokines such as TNF-α, IL-6 and IL-8 in cultured human KCs, forming a positive feedback loop between IL-36 and Th17 cytokines [16]. The increased gene expression of IL-36 is also found to be correlated with Th17 cytokines in the skin lesions of psoriatic patients. [16]. These studies have indicated a pathogenic role of IL-36 cytokines in psoriasis by driving Th1 and Th17 responses.

### Roles of IL-36 cytokines in inflammatory diseases

Over the past two decades, IL-36 cytokines have been implicated in the development of various human diseases. With a recognized central role in regulating inflammation, IL-36 cytokines mediate many inflammatory diseases, including skin diseases, inflammatory arthritis and pulmonary disorders. Available evidence suggests that IL-36 cytokines contribute to the pathogenesis of human diseases owing to their unique functions exerted between the agonists and antagonists.

### IL-36 in psoriasis

Psoriasis, as a chronic inflammatory skin disorder affecting approximately 2% of the world's population, is considered as a T-cell driven disease. Both Th 1, Th17 cells and γδ T cells have been demonstrated to participate in psoriasis pathogenesis. Dermal γδ T cells, as one of the major drivers of psoriasis pathology, are the major source of IL-17A in the skin upon IL-23 stimulation [34–35]. It has been shown that the psoriatic skin lesions highly express IL-36 cytokines whereas IL-36 agonists activate DCs and play a role in polarizing T-helper responses [24]. IL-36α, IL-36β and IL-36γ are highly expressed in skin and are involved in the pathogenesis of inflammatory skin disease psoriasis, whereby IL-36γ could be activated by cathepsin S expressed in epithelial cells, while the antagonists IL-36Ra or IL-38, another potential IL-36 inhibitor, limit uncontrolled inflammation [25–26]. Animal studies using IL-36R-deficient (*Il36r*^-/-^) mice showed that deficiency of IL-36Rresulted in reduced dermal IL-17A-producing γδ T cells and ameliorated psoriasiform dermatitis in imiquimod-induced mice [35]. The critical role of IL-36 cytokines in regulating skin inflammation is also underscored by the striking evidence that transgenic mice overexpressing IL-36α in basal KCs spontaneously exhibit skin lesions similar to psoriasis [3]. Moreover, IL-36Ra deficiency in IL-36α transgenic mice results in exacerbation of the skin manifestation, further validating the antagonistic effect of IL-36Ra on skin inflammation induced by IL-36 agonists [3]. In human psoriasis skin lesions, IL-36 gene expression is positively correlated with Th17 cytokines. The IL-36 agonists can promote the production of pro-inflammatory mediators IL-17A, IL-22, IFN-γ, TNF-α, IL-6, IL-8 and also themselves in KCs [16]. Furthermore, IL-36 agonists potently promote human KCs to express various chemokines such as CCL1-5, CCL17, CCL20, CCL22, and CXCL8 *in vitro* [36]. Intradermal injections of IL-36α lead to substantial local inflammation characterized by chemokine expression, leukocyte infiltration and acanthosis of mouse skin, further supporting a role of IL-36 in facilitating immune cell recruitment to inflamed skin [36]. In line with this evidence, patients with mutations in the IL-36RN gene encoding a nonfunctional IL-36Ra protein suffer from severe generalized pustular psoriasis [37], whereas anti-TNF-α therapy in patients with psoriasis shows significantly improved outcomes, which is associated with decreased levels of IL-36 cytokines in the skin lesions [25]. Thus, available results suggest that IL-36 cytokines actively regulate skin inflammation *via* activating KCs and mediating DC-T cell interaction, which result in tissue infiltration, cell activation and abnormal proliferation, contributing to major characteristic hallmarks of human psoriasis.

### IL-36 in arthritis

PsA is the major comorbidity of psoriasis. Approximately 20-30% of psoriasis patients develop PsA, in which skin lesions classically precede joint symptoms [38]. The genomic profiling of IL-17A, IFN-γ and TNF-α in PsA synovium shows much stronger correlation with IL-36 gene expression in PsA skin than other forms of arthritis [39]. In addition, the expression of IL-17 receptor (IL-17R) in the synoviocytes of PsA patients is significantly increased compared to that in synoviocytes of osteoarthritis patients, with concomitant augment in the number of Th17 cells in the synovial fluid, peripheral blood and skin tissue of PsA patients [39]. In the synovial tissue, IL-36α, mainly detected in CD138-positive plasma cells, was expressed at significantly higher levels in synovium of PsA and RA than that in OA. No differences were observed for the expression levels of IL-36R and IL-36Ra between PsA, RA and OA [5]. IL-36α links plasma cells to inflammatory cytokine production such as IL-6 and IL-8 by synovial fibroblasts, which may represent a key link between IL-36 and the induction of synovitis.

Currently, the role of IL-36 cytokines in RA is still controversial. In the synovium of CIA mice and RA patients, IL-36α, IL-36β, IL-36γ, IL-36Ra and IL-38 are all upregulated and correlated with the expression of IL-1β, CCL3, CCL4 and M-CSF, but not with Th17 cytokines [30]. In addition, IL-36R blocking antibody treatment does not modify clinical onset and pattern of disease in arthritic mice. Moreover, the histological features of TNF-induced arthritis have not been changed by blocking IL-36 signaling pathways [40]. Furthermore, the disease severity in collagen-induced arthritis (CIA) mice is not attenuated by neutralizing anti-IL-36R antibody [41]. Thus, these findings suggest that IL-36 signaling is not essentially involved in the development of experimental autoimmune arthritis.

### IL-36 in other inflammatory diseases

IL-36 family cytokines have emerged as important pro-inflammatory mediators in many inflammatory diseases. As summarized in Table 2, Recent studies show that IL-36 cytokines are produced by various cell types such as KCs, CD68^+^ macrophages, DCs and CD79α^+^ plasma cells in skin, synovium, joints and colonic mucosa tissues [30]. Plasma concentrations of IL-36α and IL-36γ are significantly increased in active SLE patients compared with healthy subjects, which are positively correlated with SLE disease activity and elevated plasma IL-10 concentration [31]. In patients with primary Sjögren's Syndrome, IL-36α levels are significantly increased in the blood and salivary glands, which are correlated with the serum levels of IL-17A and IL-22 [32]. In addition, recent studies have found that IL-36α and IL-36β induce neutrophil influx in mouse lungs and act as pro-inflammatory cytokines in the lung *in vivo* [47–48]. Levels of IL-36α and IL-36γ are significantly elevated in the colonic mucosa of patients with inflammatory bowel disease (IBD) and particularly, in ulcerative colitis [3]. Moreover, the elevated IL-36 expression is mirrored in the inflamed colonic mucosa of mice, whereas IL-36R deficiency results in reduced disease severity and decreased infiltration of innate inflammatory cell to the colon lamina propria in an acute dextran sulfate sodium (DSS)-induced model of colitis [6]. In patients with Crohn's disease, IL-36γ induces TNF-α expression in KCs and sustains a self-amplifying pro-inflammatory loop with IL-17C by inducing its own expression and IL-17C [5]. In CIA mice and RA patients, levels of IL-36α, IL-36β and IL-36γ expression are enhanced in inflamed joints [30]. These findings suggest that IL-36 cytokines play important roles in inflammatory diseases.

**Table 2:** IL-36 cytokine expression in inflammatory diseases

Diseases categories	IL-36 isoforms	Expression tissues	references
Psoriasis	IL-36α, β and γ	skin	[14, 42]
PsA	IL-36a, IL-36Ra	synovium	[13]
RA	IL-36α, β and γ	synovium	[43]
SLE	IL-36α, IL-36γ	blood	[44]
pSS	IL-36α,IL-36Ra, IL-36RA	labial salivary glands	[45]
IBD	IL-36α, IL-36γ	colonic mucosa	[1, 46]
ILD	IL-36α, IL-36γ	lung	[47, 48]
Obesity	IL-36α	adipose tissue	[49]

## CONCLUSIONS

**Figure 1:**
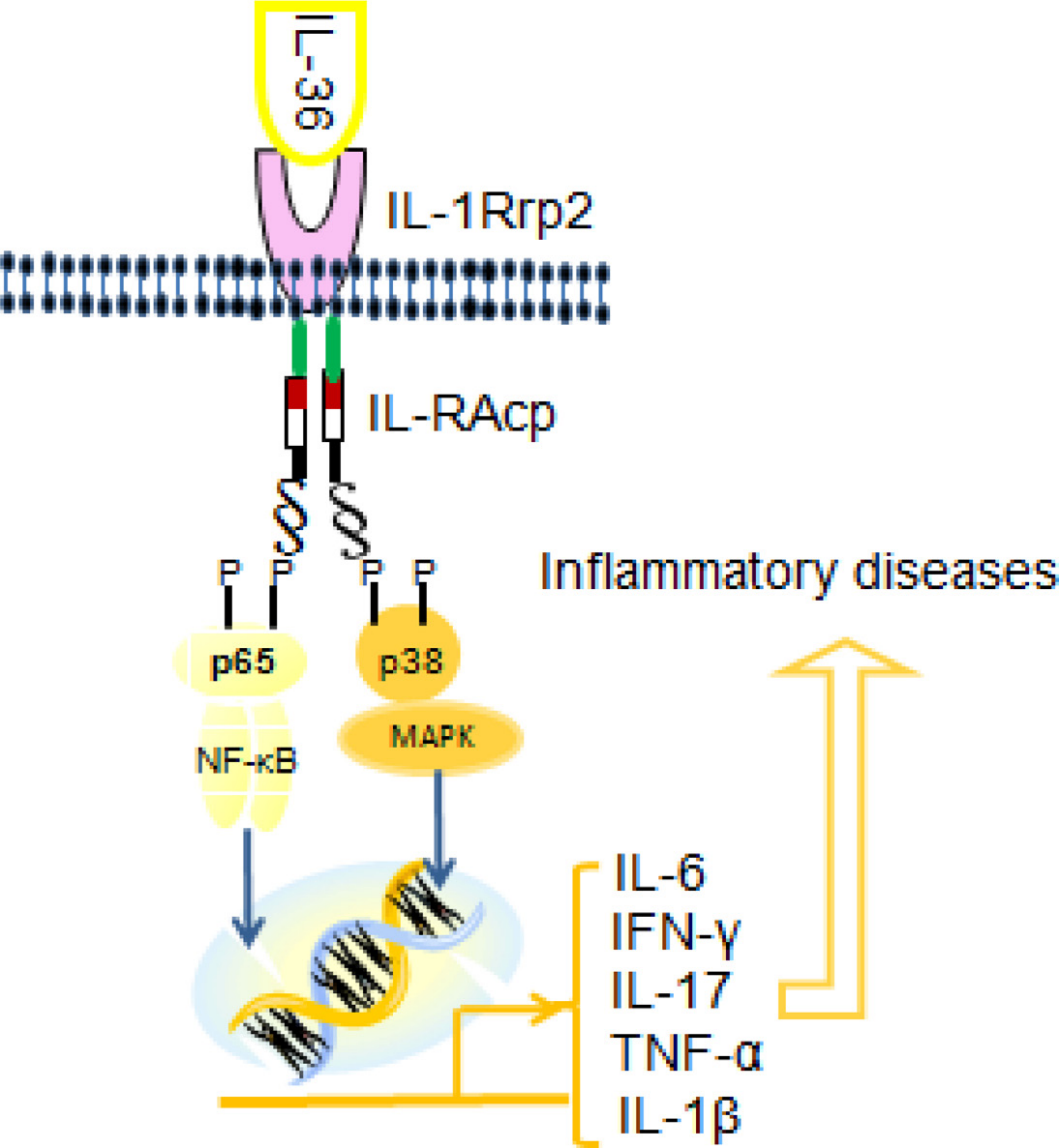
The signaling pathway of IL-36 cytokines

Both IL-36 cytokines and IL-36R signaling pathway share key features of the IL-1 and IL-1R pathway. However, the disease manifestations caused by mutations of *IL1Ra* and *IL36Ra* genes in humans suggest that the functions of these two genes have non-redundant properties. Furthermore, IL-36R is expressed in a more-restricted way than IL-1R and shows a tendency to regulate tissue-specific inflammatory responses. Therefore, IL-36 cytokines are not simple surrogates of IL-1. Instead, they are evolved to regulate tissue specific immune responses. During recent years, much has been learned about IL-36 cytokines and their role in inflammation As showed in Figure 1. Although Th17 cytokines (IL-17A and IL-22) and Th1 cytokines (IFN-γ and TNF-α) are known to play a key role in the pathogenesis of inflammatory diseases. IL-36 potently induces the production of TH1 and Th17 cytokines, which in turn enhances the function of IL-36 cytokines in an autocrine manner to form an amplification loop. Apart from their crucial role in regulating skin inflammation, IL-36 cytokines also show the important functions in inflammatory conditions of lung tissue, joint synovium and colonic mucosa tissues to promote inflammation in the skin lesion, arthritis and bowel diseases. However, further investigations are needed to elucidate the molecular mechanisms underlying their biological functions. In terms of clinical implications, future elucidation of the functions of IL-36 family cytokines in disease pathogenesis may facilitate the development of therapeutic targeting of these cytokines for the treatment of inflammatory disorders.
